# A possible role for the endocannabinoid system in the neurobiology of depression

**DOI:** 10.1186/1745-0179-3-25

**Published:** 2007-11-19

**Authors:** Gino Serra, Walter Fratta

**Affiliations:** 1 Dipartimento di Scienze del Farmaco Università di Sassari Via Muroni 23/A 07100 Sassari, Italy; 2 Dipartimento di Neuroscienze " B B Brodie " Università di Cagliari Cittadella Universitaria 09042 Monserrato (Cagliari), Italy

## Abstract

The present review synthetically describes the currently advanced hypotheses for a neurobiological basis of depression, ranging from the classical monoaminergic to the more recent neurotrophic hypothesis. Moreover, the Authors review the available preclinical and clinical evidence suggesting a possible role for the endocannabinoid system in the physiopathology of depression. Indeed, in spite of the reporting of conflicting results, the pharmacological enhancement of endocannabinoid activity at the CB1 cannabinoid receptor level appears to exert an antidepressant-like effect in some animal models of depression. On the contrary, a reduced activity of the endogenous cannabinoid system seems to be associated with the animal model of depression, namely the chronic mild stress model. Moreover, a few studies have reported an interaction of antidepressants with the endocannabinoid system. With regard to clinical studies, several authors have reported an alteration of endocannabinoid serum levels in depression, while post mortem studies have demonstrated increased levels of endocannabinoids associated to a concomitant hyperactivity of CB1 receptor in the prefrontal cortex of suicide victims. No clinical trials carried out using cannabinoids in the treatment of affective disorders have been published to date, although anecdotal reports have described both antidepressant and antimanic properties of cannabis as well as the ability of cannabis to induce mania that has also been documented. These findings are discussed, leading us to conclude that, although data available are sufficient to suggest a possible involvement of the endogenous cannabinoid system in the neurobiology of depression, additional studies should be performed in order to better elucidate the role of this system in the physiopathology of depression.

## Introduction

The present paper provides a synthetic review of the current neurobiological hypotheses of depression, taking into account preclinical and clinical evidence suggesting a possible involvement of the endogenous cannabinoid system in the physiopathology of depression.

Indeed, pharmacological manipulations of the endocannabinoid system have elicited antidepressant-like effects in animal models of depression. Moreover, some animal models of depression seem to be associated to alterations in the endocannabinod system.

Although no clinical trials performed using cannabinoids in the treatment of affective disorders have been published to date, anecdotal reports have described both antidepressant and antimanic properties of cannabis. However, cannabis abuse has been associated with the induction of psychosis and with the worsening of the course of manic-depressive disorders.

Finally, several studies have reported an interaction between antidepressants and the endocannabinoid system. Other studies have suggested that depression might be associated with alterations of endocannabinoid serum levels.

## Current hypotheses on the neurobiology of depression

The neurobiological hypotheses of depression are essentially based on the mechanism of action of antidepressant drugs.

The first hypothesis was proposed more than 40 years ago, following the serendipitous discovery of the antidepressant effect of monoaminoxidase inhibitor (MAOI) and imipramine. This hypothesis (the monoamine hypothesis of depression) postulates that depression is associated with a reduced monoaminergic transmission, in particular noradrenaline (NA) and serotonin (5HT) in the CNS [1].

The monoamine hypothesis of depression has led to the development of the more recent antidepressant drugs, namely the selective serotonin reuptake inhibitors (SSRI) and the selective noradrenaline reuptake inhibitors (SNRI). According to the monoamine theory of depression, these drugs are capable of increasing serotonin or noradrenaline levels in the synaptic cleft by inhibiting their presynaptic reuptake [2].

Although the numerous investigations aimed at demonstrating a monoaminergic deficiency in depressed patients have reported conflicting and inconclusive results[3], in our opinion the updated monoamine hypothesis [4-7] still constitutes a fundamental basis for the development of new antidepressants.

However, the above theory is not able to provide any explanation for the clinical observation that the therapeutic action of these drugs is manifested only following several weeks of treatment, while an increased monoaminergic transmission is induced immediately.

This discrepancy has generated the concept that the increase in monoaminergic transmission is manifested initially, but is not sufficient to exert an antidepressant effect. The therapeutic action of these drugs is likely associated to the neurobiological effects induced following chronic administration [8].

This consideration has led researchers to investigate the effects induced by long-term treatment with antidepressants.

Long-term administration of antidepressants is capable of modifying both the number and sensitivity of different monoaminergic receptors [8]. A detailed description of these results is beyond the scope of the present review.

It has moreover been demonstrated, by both our group and other authors [9] that chronic treatment with various antidepressants, including electroconvulsive therapy (ECT) and SSRI, produces an increase in the activity of the mesolimbic dopaminergic system, which plays an essential role in the rewarding mechanism shown to be impaired in depression [10]. These observations suggest that depression, and in particular several symptoms of depression such as anhedonia and lack of motivation, may be caused by a deficiency in mesolimbic dopaminergic transmission [10], the reinstatement of which is elicited by chronic antidepressant treatment.

However, it has been postulated that psychotic depression might be associated with an increased dopaminergic transmission, since patients may be treated successfully with the combination of antidepressants and antipsychotics [11].

More recently, clinical evidence has been reported indicating that hippocampal volume is reduced in depression [12,13].

Neuroimaging studies have reported that reduction in hippocampal volume is correlated with multiple episodes of depression in untreated patients [12,13], whereas patients treated with antidepressants do not display any loss in hippocampal volume [14,15]. These data suggest that depression might be associated with hippocampal cellular loss and/or atrophy that can be counteracted by adequate antidepressant treatments.

This hypothesis seems to be supported by recent preclinical studies, demonstrating that chronic antidepressants promote neurogenesis, while animal models of depression show decreased cell proliferation and neurogenesis.

Indeed, it has been reported that chronic antidepressants, including ECT, increase cell proliferation and neurogenesis [15], the latter being essential in producing an antidepressant like effect in animal models of depression [16].

On the contrary, acute and chronic stress decrease cell proliferation and neurogenesis [17], an effect reversed by antidepressants[17].

With regard to the mechanism by means of which antidepressants produce neurogenesis, the most convincing hypothesis suggests that antidepressants elicit such an effect by increasing the levels of neurotrophic factors such as Brain-Derived Neurotrophic Factor (BDNF)[15,18,19]. Accordingly, long-term antidepressant treatment has been shown to increase BDNF protein and mRNA levels [19-21] and reverse the stress-induced decrease in BDNF [21,22]. Exogenous administration of BDNF displays antidepressant-like effects in several animal models of depression [23]. It may be suggested that [18] long-term antidepressant treatment up-regulates cAMP pathway [24], in turn activating the transcription factor cAMP response element binding protein (CREB)[25], increasing BDNF gene expression [15,26,27].

Stress plays a key role in precipitating depression; indeed, it has been postulated that the disorder may be induced by means of a mechanism possibly related to an increased corticosterone secretion subsequently leading to a decrease in neurogenesis.

This same mechanism may also be implicated in depression associated to hyperactivity of the Hypothalamic-pituitary-adrenal (HPA) axis due to an impaired feed-back mechanism[28,29]. Indeed, the majority of depressed patients obtain abnormal results on the dexamethasone suppression test. This up-regulation produces an increase of glucocorticoid secretion which may be responsible for the decrease in cell proliferation [30-32]. Accordingly, animals lacking glucocorticoid receptors (GR) display an impaired feed back mechanism and are more susceptible to the stress-induced depressive behaviour [33].

Moreover, the exogenous administration of glucocorticoids produces a decrease in cell proliferation and neurogenesis [30-32]. Finally, hippocampal volume loss, a phenomenon that can be partially reversed once cortisol levels decrease [34], has been observed in Cushing's syndrome.

## The endocannabinoid system

A detailed description of the endocannabinoid system is beyond the scope of this paper. Thus, in this section we briefly describe those components of the endocannabinoid system that act as targets for the pharmacological interventions aimed at determining the activity of the endocannabinoid system.

The term "endocannabinoid system" refers to the recently discovered neuromodulator system comprising cannabinoid receptors (which represent the receptors of Tetrahydrocannabinol (THC), the major active component of cannabis) and their endogenous ligands.

To date, two types of cannabinoid receptors have been identified: CB1 and CB2 receptors [35-38]. These receptors belong to the superfamily of G protein coupled receptors [35,36], the CB1 receptor is widely distributed in the terminals of neurons[39], while the CB2 receptor is extensively expressed throughout the immune system[40]. However, it has recently been reported that these receptors are present also in the brain [41,42].

The activation of cannabinod receptors inhibits cAMP production via its coupling to Gi protein [35,36].

However numerous other signal transduction mechanisms associated with cannabinoid receptors have been described [43].

The main endogenous ligands (endocannabinoids) of cannabinoid receptors are anandamide [44] and 2-AG (2-arachidonoylglycerol) [45,46]. 2-AG is a full agonist at both CB1 and CB2 receptors but shows less affinity than anandamide for both receptors [47]. Anandamide is a partial agonist at both CB1 and CB2 receptors, displaying a higher affinity for the CB1 receptor [38]. Endocannabinoids are arachidonic acid derivatives conjugated with ethanolamine or glicerol. Anandamide is formed by a phospholipid precursor, the N arachidonylohosphatidyl ethanolamide (NAPE), and its release from NAPE is catalyzed by a specific phospholipase D (PLD) [48]. The 2-AG is a monogliceride synthesized by a phosphatidyl inositol specific phospholipase C(PLC) [40,47].

Following the release of endocannabinoids, these compounds exert an action on cannabinoid receptors and are rapidly inactivated by uptake and degradation [49]. Endocannabinoid uptake is mediated by a transporter facilitating the uptake of both anandamide and 2-AG [50].

The degradation of endocannabinoid is achieved by means of two specific enzymes: the fatty acid amide hydrolase (FAAH) [51] and the monoacylglyceride lipase (MAGL) enzymes [52]. FAAH degrades anandamide, whereas the MAGL degrades 2-AG.

Various compounds interacting at different phases of endocannabinoid transmission are available to better study the role of endocannabinoids in numerous pathological and physiological conditions. Accordingly, processes can be carried out by substances acting as agonists or antagonists at CB receptors, by drugs inhibiting endocannabinoid transporters and by others inhibiting FAAH activity. Moreover, very useful tools to study the physiological and physiopathological role of the endocannabinoid system are the CB1 receptor and FAAH Knockout (KO) mice.

Among the numerous of functions modulated by the endocannabinoid system [53], the control of emotions and the regulation of motivational behaviour appear to be of particular importance for the possible implication of this system in the pathogenesis of mental disorders such as drug addiction, depression, anxiety, and psychoses [54-58].

## Effect of different pharmacological manipulations of the endocannabinoid system in animal models of depression

Studies aimed at investigating the role of the endocannabinoid system in the physiopathology of depression have demonstrated how both pharmacological activation of the endocannabinoid transmission [59] and blockade of CB1 receptors [60,61] produce an antidepressant-like effect in animal models of depression, that are predictive of antidepressant activity in humans.

Possible explanation for this discrepancy will be discussed.

Hill and Gorzalka [62] have demonstrated that direct or indirect stimulation of CB1 receptor activity exerts antidepressant-like activity in the rat forced swimming test. Indeed, they observed that the administration of the uptake inhibitor AM404, the CB1receptor agonist HU210 and oleamide (which seems to be a competitive inhibitor of FAAH [62]), display an antidepressant-like effect in the rat forced swimming test similar to that observed following the administration of the classical antidepressant desimipramine (DMI). These behavioural effects are antagonized by the administration of AM251, a CB1 receptor blocker, suggesting that the stimulation of the latter receptor leads to onset of the antidepressant-like effect.

According to this hypothesis, it has been reported that URB597, a potent FAAH inhibitor [63], elicits an antidepressant-like response in the mouse tail suspension test and in the rat forced swimming test. These effects are antagonized by the administration of SR141716A, a CB1 receptor blocker, further suggesting that the activation of these receptors results in an antidepressant-like effect. Moreover, the antidepressant-like effect produced by URB597 was also observed after 4 days of sub-chronic treatment with the drug.

The administration of URB597 in the rat forced swimming test induced a behavioural pattern similar to that observed with fluoxetine (decreased floating, increased swimming but no affect on struggling) but at variance with that displayed following DMI (increased struggling but not swimming).

The behavioural effects elicited by URB597 appear to be manifested following the stimulation of CB1 receptors (attenuated by the CB1 receptor blocker SR141716A) by anandamide.

Indeed, URB597 increases anandamide levels in the hippocampus, prefrontal cortex (PFC) and midbrain, but does not affect 2-AG levels. Moreover URB597 increases the firing rate of 5HT neurons in the Dorsal raphe nucleus (DRN), an effect becoming increasingly evident after repeated administration and which is antagonized by SR141716A. Repeated administration of URB597 also increases 5HT release in the hippocampus. On the other hand, URB597 produces a slow increase in the activity of noradrenergic neurons in the locus coeruleus, but does not affect noradrenaline release. These observations have led the authors to suggest that the antidepressant-like activity of URB597 may be mediated by the increase of 5HT and NA transmission.

However, this interpretation contrasts with the behavioural observation made in the forced swimming test, in which URB597 behaves in a similar fashion to fluoxetine, a SSRI.

The chronic administration of URB597 exerts an antidepressant-like effect also in the chronic mild stress model of depression with a concomitant increase of anandamide levels in the midbrain, striatum, and thalamus [64].

However, recent reports refer to how URB597 failed to elicit an antidepressant-like effect in the mice tail suspension test and in the forced swimming test. Likewise, no antidepressant-like effect was manifested in FAAH-KO mice [65]. However, this discrepancy may be explained by the different experimental conditions used. In fact when Lichtman's group performed experiments with altered experimental conditions such as altered ambient light and increased sample size they observed an antidepressant-like effect in the mice tail suspension test of URB597 and FAAH KO-mice[65].

Antidepressant-like properties have also been observed following administration of ACEA (arachidonyl 2 chloroethylamide) [66], a selective CB1 receptor agonist capable of eliciting an antidepressant-like effect in the mice forced swimming test and in the head twitch response to L5HTP.

Long term treatment with the CB1 agonist HU210 promotes neurogenesis in the hippocampus of adult rats and elicits antidepressant-like behaviour in the forced swimming test [67]. These effects are blocked by the CB1 receptor antagonist AM281 and by hippocampal irradiation which blocks neurogenesis.

These observations suggest that the antidepressant-like effect displayed by the CB1 receptor agonist is related to the promotion of hippocampal neurogenesis.

Interestingly, a similar effect has been reported after long term administration of different classes of antidepressants [15,16].

Taken together, the above observations suggest that the activation of CB1 receptors through direct administration of agonists or subsequent to increased endocannabinoid levels at CB1 receptors may produce a potential antidepressant effect.

However, this hypothesis is in contrast with the findings that also the blockade of CB1 receptors produces an antidepressant-like effect in animal models of depression.

The administration of AM251 has been reported to decrease immobility in the mice tail suspension test in a dose-dependent manner [68], an effect similar to that observed after DMI.

On the contrary, the administration of the CB1 receptor agonist CP55940 increases immobility in the mice tail suspension test and counteracts the effect of AM251[68].

The latter compound also elicits an antidepressant like effect in the rat forced swimming test [68] whilst producing no effect in CB1 receptor KO mice.

This previous observation led the authors to hypothesize that the antidepressant-like behavioural effect elicited by the CB1 receptors antagonists is strictly associated to the presence of the CB1 receptors.

In accordance with the findings of Shearman et al [68], an additional study reported how SR141716A increases monoamine release in the prefrontal cortex and reduces immobility in the mice forced swimming test when administered at the relatively high dose of 3 mg/Kg but not at the low doses of 0,3 mg/Kg and 1 mg/Kg [69].

A similar effect has been observed by Griebel et al [70]. Indeed, the administration of SR141716A at the dose of 3 and 10 mg/Kg does elicit an antidepressant-like effect in the rat forced swimming test similar to that observed after the administration of 30 mg/Kg of fluoxetine.

Finally, recent reports have disclosed that AVE1625, a novel cannabiniod CB1 receptor antagonist [71], produces an antidepressant-like response in the mice forced swimming test [71].

In an attempt to provide an explanation for these conflicting results, further investigations aimed at evaluating the effect of the increase of CB 1 activation and CB1 receptor blockade under identical experimental conditions and assessing the activation and blockade of CB1 receptors in different animal models of depression should be performed.

It has been suggested that the antidepressant-like effect elicited by SR141716A may be produced following activity at the level of an as yet uncharacterised central cannabinoid receptor[59].

Accordingly it has been reported that SR141716A stimulates neurogenesis also in CB1 KO mice [72], indicating the involvement of a non CB1receptor. In fact, SR141716A administered to Vanilloid receptor (VRI)-KO mice failed to induce neurogenesis [72], suggesting that rather than the neurogenic effect of SR141716A being mediated by a non CB1 receptor, it is more likely regulated by the stimulation of VRI receptors.

In addition, the possibility cannot be ruled out that the efficacy of SR141716A in the forced swimming test could be due to its ability to stimulate locomotor activity [73]. On the contrary, CB1 receptor agonists have been reported to reduce locomotor activity, suggesting that their effect in the forced swimming test cannot be considered a false positive secondary to the increased motor activity [74].

On the other hand, CB1 KO mice have been reported to display depressive-like behaviour [55].

## Endocannabinoid system in animal models of depression

The majority of studies aimed at investigating the role of endocannabinoid system in animal models of depression report data suggesting that these models are associated with a decrease in activity of the endocannabinoid system.

CB1 KO mice have been shown to be more susceptible to developing depressive-like behaviour according to the chronic mild stress model of depression [55]. This animal model of depression measures the preference for a sucrose solution following chronic exposure to mild stress. A reduction in this preference is considered a symptom of animal anhedonia. Thus, the decrease in sucrose intake by CB1 KO mice suggests that the animals may posses a higher sensitivity towards developing anhedonia, a core symtom of depression.

Accordingly, blockade of the endocannabinoid system activity reduces the reinforcing properties of natural and artificial stimuli [55,75], an effect that should result in anhedonia.

The CB1 receptor KO mice are more susceptible to the neurotoxic effect of kainic acid and display a marked decrease in adult neurogenesis [72].

Furthermore, exposure to chronic stress is capable of down-regulating the endocannabinoid system by reducing CB1 receptor density and 2-AG levels in the hippocampus [76].

However it has been reported that blockade of CB1 receptors with SR141716A reduces the depressive-like behaviour observed after chronic exposure to mild stress [70].

Contrary to observations made as to the effect of different pharmacological treatments in animal models of depression, only one report [70] has demonstrated a decrease in depressive behaviour produced by the administration of SR141716A following chronic exposure to mild stress. Nevertheless the dose of SR141716A used in this experiment was relatively high (10 mg/Kg per os).

Furthermore, CB2 receptors appear to be involved in the physiopathology of depression. Indeed, it has been reported that the expression of CB2 receptors is increased in the mouse brain after 5 weeks of chronic mild stress [42].

## Effects of antidepressant drugs on the endocannabinoid system

To date very few studies have been published with regard to the effect produced by the currently available antidepressant drugs on the endocannabinoid system.

Repeated administration of fluoxetine causes a decrease of cannabinoid CB1 gene expression in the caudate-putamen [77]. The authors suggest that fluoxetine may inhibit anandamide uptake [77], thus increasing the availability of the endocannabinoid at the level of the CB1 receptor; increased activation of the receptor would likely result in down-regulation of cannabinoid CB1 receptors gene expression [77].

On the contrary, it has been reported that chronic fluoxetine causes an up-regulation of CB1 receptors in the Prefrontal cortex (PFC), [78,79], chronic DMI up-regulates CB1 receptors in the hypothalamus and hippocampus [78,79], tranylcypromine (a MAOI) decreases the levels of anandamide in the PFC, hippocampus and hypothalamus, and increases CB1 receptor binding in PFC and the hippocampus, but not in the hypothalamus [78,79].

The results suggest that tranylcypromine induces hypo-activity of the endocannabinoid system in the hypothalamus, but, similar to fluoxetine and DMI, produces an up-regulation of CB1 receptors in other brain areas.

Moreover, the observation that chronic fluoxetine activates G protein signal transduction coupled to CB1 receptors at different levels [80] adds further support to the hypothesis that fluoxetine activates the endocannabinoid system.

A recent study published by Hill et al [81] demonstrated that chronic administration of DMI does not modify endocannabinoid levels in the PFC, hippocampus, hypothalamus, or amygdala, but increases CB1 receptor density in the hippocampus and hypothalamus.

Moreover, rats subjected to a 5 minutes swimming test displayed an increase in corticosterone levels and the induction of c-fos in the Paraventricular nucleus(PVN) of the hypothalamus.

Chronic DMI counteracts these stress-induced effects on the HPA-axis. The effect of DMI is mediated by the activation of CB1 receptors being blocked by the administration of the CB1 receptor antagonist AM251.

This finding suggests that antidepressant drugs may restore the HPA-axis by stimulating CB1 receptors, thus stimulating the endocannabinoid system.

However, Gobshtis et al [82] have demonstrated that the antidepressant -like effect produced by DMI and fluoxetine in the mice forced swimming test was not antagonized by SR141716A.

This finding is in line with the observation of Sherman et al [68] who demonstrated that the antidepressant-like effect of DMI was also displayed in CB1 KO mice.

The above observation suggests the impossibility of generalising the involvement of CB1 receptor in the action of DMI, tending rather to be limited to producing an effect on the regulation of the HPA-axis.

Recently, a very complex action of electroconvulsive shock (ECS), the most powerful antidepressant treatment, on the endocannabinoid system has been reported [83]. A single administration of ECS produces a reduction in the affinity of CB1 receptors in the PFC, hippocampus, hypothalamus and amigdala, reduces anandamide levels in the PFC and hippocampus, and FAAH activity in the PFC. On the contrary, it increases the density of CB1 receptors in the amigdala. After chronic administration of 10 days ECS further reduces the anandamide levels in the PFC as well as the density of CB1 receptors, but enhances the sensitivity of CB1 receptors in the amigdala.

## Clinical studies

To date, the only clinical trials present in the literature performed using synthetic compounds capable of influencing the endocannabinoid system are those aimed at evaluating the efficacy of rimonabant in obesity [84,85].

The rationale underlying these studies was based on the observation that the blockade of CB1 receptors is associated to a reduction of reinforcing properties elicited by both natural and artificial stimuli [55,75].

A clinical study carried out by Despress et al [84] includes depression among the side effects resulting in discontinuation of treatment. This observation leads us to hypothesize that blockade of CB1 receptors may be capable of inducing depression in humans. This is more than a mere speculation if we consider that the Food and Drug Administration (FDA)'s Endocrinologic and Metabolic Drugs Advisory Committee meeting on June 13, 2007, rewieving numerous studies and post-marketing report did not recommended approval of Rimonabant mainly because of their concerns about the ability of the drug to increase the risk of depression and "suicidality"[86] This effect appears to be present not only in people with a history of depression but also in patients without history of depression, since some clinical trials with rimonabant do not include patients with history of depressive disorders, with a depressive episode or those in therapy with antidepressant drugs.

Moreover, the European Medicines Agency(EMEA)'s Committee for Medicinal Products for Human use(CHMP) on July 19, 2007 decided to include " ongoing major depression illness and/or ongoing antidepressant treatment " among the contraindications to the use of rimonabant; moreover, the administration of the drug must be stopped if depression develops[87].

To the best of our knowledge, however, no clinical studies have been performed to study mood disorders using synthetic drugs capable of interacting with the endogenous cannabinoid system. Although several anecdotal reports have been published with regard to the effects produced by cannabinoids on depression and or mania, to date, no clinical trials have been performed to investigate the therapeutic use of cannabinoids in depression and or mania [88].

The previously mentioned anecdotal reports describe both antimanic [89] and antidepressive effects[90] of cannabis. Conversely, other reports have disclosed how cannabis may induce mania [91,92] and psychosis [93], while substance abuse increases the severity of mood disorders [94]and even the risk of suicide[95].

Interestingly, Hungund et al [96] found an up-regulation of CB1 receptors associated to a concomitant increase in the CB1 receptor mediated GTP binding in the PFC of depressed suicides, suggesting an important role for enhanced endocannabinoid activity in the pathogenesis of suicide. Accordingly, Vinod et al [97] reported elevated levels of endocannabinois, CB1 receptors and CB1 receptors mediated GTP binding in the PFC of alcoholic suicides, adding further support to an involvement of hyperactivity of the endocannabinoid system in suicides [98].

Very recently, Hill et al [99] found that serum 2-AG is reduced in patients suffering from major depression, a reduction strictly correlated with the duration of the depressive episode. On the contrary, serum anandamide levels in these patients remain unchanged, whereas patients with high anxiety scores are characterised by lower levels of anandamide.

Significantly elevated serum anandamide levels were revealed in patients with minor depression. However, it must be pointed out that the same authors found (" in a second experiment ") a decrease of both anandamide and 2-AG in women affected by major depression.

Exposure of these patients to psychological stress produced an initial increase in 2AG and anandamide levels followed by a subsequent decrease.

Finally, Miller et al [100] found that 2-AG serum levels were decreased in women with major depression, this decrease being correlated with the duration of the depressive episode. On the contrary women suffering from minor depression displayed significantly high serum levels of anandamide.

On the basis of these observations, the authors suggest that modifications to the central endocannabinoid system vary in the presence of minor or major depression, the system becoming hypoactive in major depression and displaying an increased activity in minor depression.

Accordingly, Koethe et al[101] found a decrease of CB1 receptor density in the glial cells of the grey matter in the brain of post-mortem patients suffering from major depression.

## Conclusion

The preclinical and clinical data presented in the present review may suggest involvement of the endocannabinoid system in the pathogenesis of depression.

Preclinical data were derived exclusively from studies performed in animal models of depression, the majority of which used the rat forced swimming test model of depression, reporting largely conflicting results.

Indeed, both the activation and the blockade of the endocannabinoid system have been reported to produce antidepressant-like behaviour. Thus, on the mere basis of the findings reported, it is difficult to hypothesise more specifically as to the role played by endocannabiniods in the pathogenesis of depression.

Additional studies should be performed in different animal models of depression to elucidate whether an increased or decreased activity of the endocannabinoid system might exert a potential antidepressant effect and whether, in turn, depression may be associated with an enhanced or a deficient functioning of this system.

However, studies performed on the endocannabinoid system in animal models of depression, suggest an association between a reduced activity of the endocannabinoid system and depressive behaviour induced by chronic mild stress model.

However, to better support this hypothesis, also in this case, similar studies should be performed using different animal models of depression.

Studies published to date on the effect of classical antidepressants on the endocannabinoid system seem to suggest an increased transmission induced by antidepressants at CB1 receptor level although negative findings have also been reported.

Of particular interest is the finding that the stimulation of cannabinoid CB1 receptors promotes neurogenesis while CB1 KO mice show impaired adult neurogenesis.

These findings are in line with the more recently proposed theories on the neurobiology o f depression.

Clinical data available to date suggest that the endocannabinoid system might be implicated in the pathogenesis of suicide, apparently associated with a hyperactivity of the endocannabinoid system. Indeed, this observation also suggests that enhanced endocannabinoid activity might be associated with impulsive behaviour, a characteristic of suicide, rather than with depression.

Moreover, lower levels of 2-AG have been found in major depression while minor depression appears to be associated with high levels of serum anandamide.

With regard to the effect of pharmacological manipulation of the endocannabinoid system in the treatment of depression, the literature reports mainly anecdotal observations proposing both antidepressive and antimanic effects for marijuana. Habitual marijuana users tend to manifest mania, worsening of mood disorder course or psychosis, suggesting that cannabinoids may produce a stimulant effect manifested not only in the presence of euphoria but also in the presence of mania and psychosis.

In line with this hypothesis is the report that blockade of cannabinoid CB1 receptors might induce depression. Indeed, it is tempting to speculate that depression might be associated with a decrease of the endocannabinoid system while mania is manifested subsequent to a hyperactive functioning of the system. According to this hypothesis it has been recently reported that SR141716A antagonizes amphetamine-induced arousal in the Cebus monkey [102]. To further investigate this hypothesis, detailed studies are currently in progress in our laboratory with the aim of evaluating the effect of CB1 receptor blockers in animal models of mania.

To conclude, although some preclinical and clinical evidence may seem to suggest an involvement of the endocannabinoid system in the physiopathology of depression, it is undeniable that additional studies aimed at better elucidating the role of the endocannabinoid system in the neurobiology of depression are mandatory.

The latter studies should focus particularly on clarifying the role of the different pharmacological manipulation of the endocannabinoid system in different animal models of depression and mania. Furthermore, studies performed to investigate the effect of different classes of currently used antidepressants and mood stabilizers on the various stages of endocannabinoid transmission should prove to be of great interest.

Lastly, the relevance of serum levels of endocannabinoids throughout the different phases of manic depressive disorders should be ascertained in order to better clarify the role of these neuromodulatory compounds in the neurobiology of depression. Moreover, we are confident that neuroimagine studies should provide very interesting results to elucidate the role of the endocannabinoid system in the different phases of mood disorders.

## Competing interests

The author(s) declare that they have no competing interests.

## Authors' contributions

The authors contributed equally to this work
